# AI in Radiology: Navigating Medical Responsibility

**DOI:** 10.3390/diagnostics14141506

**Published:** 2024-07-12

**Authors:** Maria Teresa Contaldo, Giovanni Pasceri, Giacomo Vignati, Laura Bracchi, Sonia Triggiani, Gianpaolo Carrafiello

**Affiliations:** 1 Postgraduation School in Radiodiagnostics, University of Milan, 20122 Milan, Italy; giacomo.vignati@unimi.it (G.V.); sonia.triggiani@unimi.it (S.T.); gianpaolo.carrafiello@unimi.it (G.C.); 2 Information Society Law Center, Department “Cesare Beccaria”, University of Milan, 20122 Milan, Italy; 3 Cerba Healthcare Italia, 20139 Milan, Italy; laura.bracchi@cerbahealthcare.it; 4 Radiology and Inverventional Radiology Department, Fondazione IRCCS Cà Granda, Policlinico di Milano Ospedale Maggiore, 20122 Milan, Italy

**Keywords:** Artificial Intelligence Systems (AISs), responsibility, liability, transparency, black-box phenomenon, European doctrine, decision-making process, computernalism

## Abstract

The application of Artificial Intelligence (AI) facilitates medical activities by automating routine tasks for healthcare professionals. AI augments but does not replace human decision-making, thus complicating the process of addressing legal responsibility. This study investigates the legal challenges associated with the medical use of AI in radiology, analyzing relevant case law and literature, with a specific focus on professional liability attribution. In the case of an error, the primary responsibility remains with the physician, with possible shared liability with developers according to the framework of medical device liability. If there is disagreement with the AI’s findings, the physician must not only pursue but also justify their choices according to prevailing professional standards. Regulations must balance the autonomy of AI systems with the need for responsible clinical practice. Effective use of AI-generated evaluations requires knowledge of data dynamics and metrics like sensitivity and specificity, even without a clear understanding of the underlying algorithms: the opacity (referred to as the “black box phenomenon”) of certain systems raises concerns about the interpretation and actual usability of results for both physicians and patients. AI is redefining healthcare, underscoring the imperative for robust liability frameworks, meticulous updates of systems, and transparent patient communication regarding AI involvement.

## 1. Introduction

Artificial Intelligence (AI) presents considerable advantages in enhancing diagnostic accuracy and clinical management, driving advancements in health research and pharmaceutical innovations, and optimizing the administration of healthcare services and resources. Nevertheless, the rapid progress of Artificial Intelligence Systems (AISs) introduces significant ethical and legal challenges: it is crucial to address them to ensure a comprehensive integration in overseeing patient care [1].

To build trust and encourage the adoption of AI technologies, both the World Health Organization (WHO) and the Food and Drug Administration (FDA) are actively working to create strong governance models for AISs with the aim of developing reliable guidelines and regulatory frameworks.

To the best of the authors’ knowledge, the current literature is not comprehensive and lacks a panoramic view of the legal challenges associated with the use of AI in the radiology department. 

The incorporation of AI into everyday clinical healthcare practices is grounded in its promise to drastically reduce errors and harm, resulting in a paradigm shift where AI becomes a fundamental component of established care standards [2,3]. Nonetheless, acknowledging the potential for AI-induced errors (AI-iatrogenesis) is realistic and, in such scenarios, the question of where responsibility lies becomes particularly relevant [2]. 

Under product liability theory, patients who are harmed or injured by products that are not “*reasonably safe*” because of design flaws, poor manufacturing, or insufficient warnings have the right to sue for monetary compensation. The law specifies that makers of prescription drugs and medical devices bear liability for damages caused by these defects. The legislation mirrors the FDA’s recognition that prescription medical products inherently possess unavoidable risks, thereby necessitating physician authorization as a prerequisite prior to their utilization [4]. 

In any common-law country, legal standards of care are set by the professional group themselves [2].

In recent years, numerous studies have focused on the application of AI in healthcare, and the spotlight has increasingly turned to how artificial intelligence is transforming legal reasoning. This article aims to bridge that gap by offering an engaging narrative that presents a focused and insightful discussion, capturing the diverse issues of reasoning, judgment, and AI-driven decision-making in radiology across the legal domain in Europe.

Our question was “*Who will be responsible when there is medical radiological malpractice eventually due to the defective AI technology?*”. When AI is integrated into clinical practice, assigning accountability in instances of negative outcomes becomes both complex and critical. A preliminary multidisciplinary brainstorm with legal experts, radiology residents, and radiologists with decades of experience was conducted to assess the aim of the paper and the roles of the authors. Then, a free text search was conducted on PubMed, with the selection of articles equally performed by all residents. To reach a panoramic overview of the legal aspects of AIS application in radiology, residents conducted comprehensive research on the PubMed database. 

After identifying the relevant articles, the authors began writing the systematic review, each drafting an individual manuscript that reflected their distinct analytical perspectives. These manuscripts were then discussed and evaluated for final revisions, following collegial agreement.

In the final paper, the authors started with a focus on the significance of responsibility in jurisprudence, clarifying, with brief references, the legal concepts of *Accountability*, *Culpability*, and *Liability*. Then, the use of AI as a medical device was analyzed, delving into the concept of ‘learned intermediary doctrine’. Subsequently, the focus shifted to the “system transparency” topic and the “black box systems” theory to comprehend the AI’s decision-making process. In the end, the writers introduced the concept of “*computernalism*” as a new form of decision-making tool. In the AI era, the conventional bilateral doctor–patient relationship is evolving into a trilateral “physician-computer-patient” relationship, heralding a major transformation in healthcare. 

In this changing landscape, there is an urgent requirement for clear regulations concerning the legal responsibilities of healthcare providers integrating AI into their practices.

To conclude, the researchers decided to further emphasize the importance of legal aspects of AI in healthcare, given the undeniable advantages of its daily use.

## 2. Materials and Methods

Initially, a multidisciplinary brainstorming session with legal experts, radiology residents, and experienced radiologists was held to determine the aim of the paper. The authors then conducted some free text searches on PubMed to capture relevant keywords for a subsequent comprehensive survey. 

**Figure 1:**
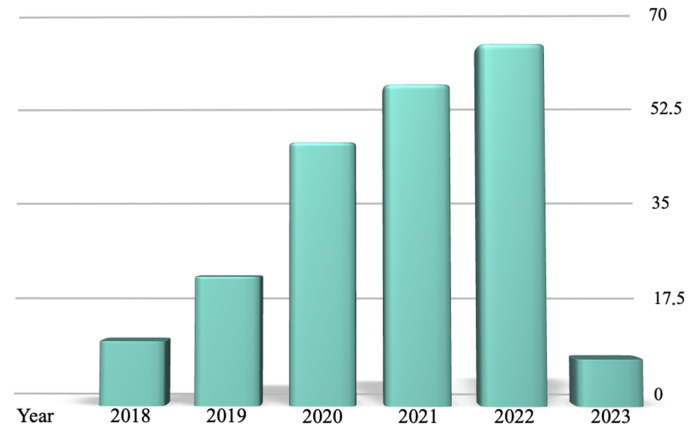
The results reported by the PubMed research over the 5 years analyzed (from 2018 to 2023), categorized by year of publication.

The comprehensive literature search employed a specific combination of keywords (“legal” AND “artificial intelligence”), restricted to a period of five years from February 2018 to February 2023. This period, as shown in Figure 1, marked a significant era of development in AI and Law research. 

To ensure the inclusion of recent and relevant studies in the evolving landscape of artificial intelligence (AI) in legal contexts, filters were added to identify articles classified as “*Reviews*” and “*Systematic Reviews*”. 

A total of 182 papers were initially identified and subjected to a rigorous analysis by three independent reviewers. 

The focus of this research was specifically tailored to examine the legal aspects related to AI-enabled decision-making tools in radiology, excluding studies that addressed other healthcare domains outside the radiological field. This decision was collectively agreed upon by all authors, emphasizing the importance of understanding the legal challenges and opportunities inherent in the use of AI within radiology. Articles published in languages other than English and articles available only in their abstract form were excluded. Discrepancies regarding the relevance and quality of the papers were resolved through multidisciplinary discussion to achieve a unanimous consensus. This collaborative approach facilitated the exclusion of articles that did not meet the predetermined criteria or were deemed irrelevant to the core objectives of this review.

**Figure 2:**
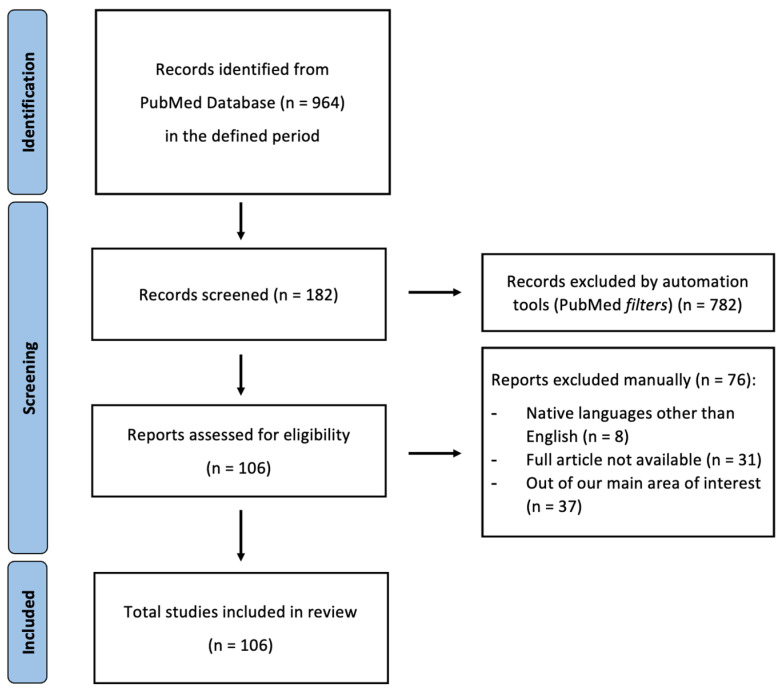
PRISMA 2020 flow diagram for updated systematic reviews; from [5].

Authors rigorously adhered to the Preferred Reporting Items for Systematic Reviews and Meta-Analyses (PRISMA) guidelines to ensure a meticulous approach, as shown in Figure 2.

In instances of ambiguity or disagreement, the reviewers conducted consultations to agree on excluding certain papers, thus safeguarding the integrity and focus of the paper. 

Upon completion of the evaluative phase, each reviewer drafted an individual manuscript that reflected their distinct analytical perspective on the collected data. 

These preliminary manuscripts were then discussed in a series of meetings, leading to the integration of diverse viewpoints into a cohesive final paper.

**Figure 3:**
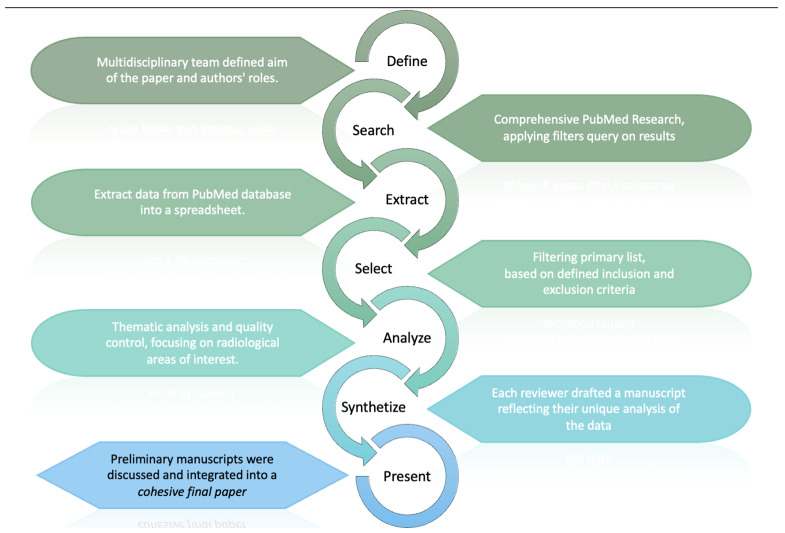
The review process, which began with defining aims and roles. PubMed research, data extraction, and filtering led to thematic analysis of legal aspect of AISs in radiology. Reviewers then unified their drafts into a cohesive paper.

This methodology, schematized in Figure 3, facilitated an in-depth examination of the current legal considerations surrounding the application of AI in radiology, providing a multifaceted analysis of the topic. 

The intrinsic limitations of this study pertain to the use of a single database with highly specific keywords. Additionally, we intentionally confined our analysis to European jurisdictions, excluding global legislation (such as that of China or the USA), and opted not to present the topic of “AI explainability”. These decisions were made to maintain a focused and manageable scope for our paper, as incorporating these broader topics would have significantly expanded the discussion and detracted from the main point.

## 3. Discussion

Artificial intelligence can exhibit a broad spectrum of performance, shifting from highly advanced cognitive capabilities to markedly basic behavior without warning. Regardless of efforts to mitigate AI bias, intrinsic limitations persist within all AI constructs. The ability to assign responsibility to the developers or operators is potentially compromised by systems that can function independently and learn new behavioral patterns. The deployment of AI technologies may result in an accountability void, with no identifiable party to hold responsibility, thereby significantly constraining our capacity to attribute fault and assume responsibility for decisions made [6]. 

Responsibility within jurisprudence is classified into three main categories: (1) **Accountability**, (2) **Culpability**, and (3) **Liability**.

The term “*accountability*” refers to the capability of a system to provide an explanation for its actions, encompassing a detailed account of its input(s), internal processes, and output(s). A person is deemed “*culpable*” for a committed punishable action if this action was either intentionally and freely undertaken, or if the individual failed to take necessary measures to prevent the act. 

Given these definitions, an AI program should be “accountable” but can not be considered “culpable” for its actions, as it lacks free will, consciousness, and the capacity for autonomous decision-making.

Accountability and culpability are prerequisites for assigning liability, but traditional rules of liability law do not fit when dealing with unpredictable machines. Essentially, humans do not have much control over the decisions these machines make [7]. 

On the other hand, actions always entail accountability: AIS cannot be held liable, and radiologists have to bear the burden through their deliberate choice to integrate such technologies into their practice.

Any action invariably results in the assignment of responsibility, not to the machine system itself, but to the individuals who developed it and those who deploy it [8].

Radiologists stand as the autonomous and informed mediators between software suggestions and patient welfare yet find themselves facing the challenge of being accountable for AI’s advice, with only limited control over its output [9].

In cases of medical errors, the responsibility primarily remains with the physician, given that he/she conducted or oversaw any clinical duty. Moreover, there is an ongoing legal debate about potentially allocating some degree of liability to the developers of these machine learning applications. When physicians choose to disregard predictions made by machine learning, legal standards force them to demonstrate that their decisions are rational and align with the expected conduct of a competent practitioner in their field. While it might be proposed that machine learning tools should be dispensed from such professional standards, this issue may change as these technologies are increasingly integrated into the clinical decision-making landscape.

While it may not be essential for radiologists to comprehend the intricate mathematical computations underlying machine learning algorithms, they could benefit from understanding the types of data utilized for predictions and the importance attributed to each data category. Similarly to the interpretation of results from clinical tests, radiologists could evaluate characteristics like sensitivity and specificity when predicting disease risk or response to treatment using these tools. Additionally, several nations are working on acknowledging the patient’s right to understand the workings of machine learning technologies applied in their healthcare [10].

Traditional approaches to product liability and consumer protection might not be considered in AI-harm scenarios because of the complex interactions among physicians, machines, and patients, along with the overlapping duties of care. Regulators are forced to consider alternative, fault-neutral insurance strategies. Sung [2] suggested creating taxes for the usage of AIS so that these strategies could provide compensation to injured victims, irrespective of fault determination.

Multiple institutions, such as the World Health Organization (WHO) and the Food and Drug Administration (FDA), are undertaking initiatives to establish robust AI governance models. These efforts are directed towards the development of trustworthy AI guidelines and regulatory frameworks, with the objective of enhancing trust and promoting the adoption of AI technologies. Institutions employing AI to improve healthcare outcomes must rigorously ensure compliance with legal and safety standards to mitigate malpractice risks. This necessitates the implementation of adequate safety protocols and the correct application of AI-driven medical devices, particularly within radiology departments: these entities must not only integrate AI into their operational frameworks but are responsible for implementing proper controls (enhancing patient care without compromising standards) and applying AI-driven medical devices for their precise and intended use [11].

Indeed, as part of the quality assurance (QA) process for the system, it is prohibited to modify the intended use of a commercially available AI tool to suit a clinic’s requirements [12]. 

### 3.1. AI as a Medical Device

In the European Union (EU), AI-based medical devices are regulated by the Medical Devices Regulation (MDR), which ensures their safety, effectiveness, and clinical reliability. Under Directive 93/42/EEC, the EU defines a “medical device” as “any tool, software, material, or item used for diagnostic and/or therapeutic purpose and intended for diagnosis, prevention, monitoring, or treatment of diseases, injuries or disabilities” and “for investigation or modification of the anatomy or physiological processes and contraception control”. Its foundational operation transcends pharmacological, immunological, or metabolic modalities, although these modalities may augment its functionality [13].

When an AI-based device qualifies as a medical device under EU-MDR, it must comply with General Safety and Performance Requirements (GSPRs). Its safety and performance summary must detail its diagnostic or therapeutic use, including an assessment of its clinical effectiveness relative to alternative options available, considering its specific circumstances of usage [14]. 

According to MDR, devices must have a CE mark to ensure their unrestricted distribution and utilization within the EU. Member States are discouraged from obstructing devices that comply with these standards, yet they retain the authority to restrict certain device applications on issues not addressed by MDR [14]. 

The CE mark obligation is waived for software exclusively created and utilized within a healthcare institution, assuming it is not transferred elsewhere and no other alternative commercial device sufficiently meets specific patient needs [12].

AI manufacturers must meet strict conformity assessment, clinical evaluation, and post-market monitoring requirements to ensure device quality and reliability in the EU market. Due to the innovative nature of AI tools, manufacturers often lack a full understanding of their suitability, clinical effectiveness, and generalizability, potentially skewing expected benefits. This partial understanding might lead to inaccurate risk assessment during pre-market evaluation (either overestimating or underestimating risks). Post-market clinical follow-up may be necessary to ensure a comprehensive understanding of the device’s real-world clinical application [12].

In this context, qualified Medical Physicist Experts (MPEs) play a crucial role in assessing the safety and effectiveness of AI tools before clinical integration. They may also contribute to AI algorithm development [12].

In April 2021, the European Commission introduced the first legislative framework for AI regulation within the EU, titled “Proposal for a Regulation of the European Parliament and of the Council, establishing uniform regulations on the development, deployment, and use of AI systems, representing a significant step milestone in the AI regulation within the EU [15]. 

The proposed AI Act focuses on regulating AI systems that impact public health, safety, or human rights, establishing a framework for the assessment and management of potential risks. AI applications are required to adhere to the principles and regulations outlined in the upcoming AI Act and align with the Medical Devices Framework (MDF). The MDF consists of the Medical Devices Regulation and the In-Vitro Medical Devices Regulation and is already applicable for placing AI medical devices on the market [16,17]. 

The AI Act proposal outlined measures to ensure AI systems comply with fundamental rights, transparency, accountability, and safety standards, balancing innovation with the protection of individuals and society and building trust in AI technologies for their ethical and responsible use in daily life. Transparency has been established as a fundamental requirement for AI-integrated medical devices: a system must allow users to comprehend and apply the AI’s outputs effectively. The EP Report on AI Framework [18] already distinguished between transparency of the algorithm (knowing how algorithms work) and transparency of usage (understanding how they’re used). This difference matters because even if the main challenge in explaining AI decisions lies with the algorithms, other aspects (like the data used by AI and the actions of people involved in AI’s development, deployment, and usage) are easier to clarify and must be controlled. Thus, transparency efforts should not only focus on the technical side (such as making algorithms understandable) but also on managing data properly and clearly assigning responsibilities to those working with AI. This broader approach is a more comprehensive strategy for addressing AI transparency [1].

Additionally, the EC Proposal introduced a risk-based approach to classify AI systems into different categories based on their potential impact, imposing stricter requirements and obligations for higher-risk applications [14]. 

Recent literature highlights emerging limitations concerning the AI Act, notably its integration into existing laws like cybersecurity regulations for specific technologies, including medical device software, yet it suffers from an unclear definition of cybersecurity [19]. Furthermore, Van Kolfschooten [20] raises concerns that AIS may intensify issues such as discrimination and privacy violations, compromising fundamental values and the rights of patients. This critique centers on the European approach to adopting AIS in healthcare, which seemingly neglects the specific challenges introduced by AI—particularly in medical decision-making processes—and fails to adequately protect the vulnerability of patients.

Meszaros et al. [21] delved into the intricate relationship between the GDPR and the proposed AI Act, observing that the latter could sometimes provide a legal framework for the processing of personal data. They emphasize the necessity of harmonizing AI and data protection legislations within the EU to secure an innovative future, highlighting the challenge and importance of integrating data protection with AIS, especially in healthcare and medical research.

Moreover, Ebers et al. [22] identified two primary challenges associated with AIS: the risk of overregulation due to a wide-ranging definition of AI and the potential danger of under-regulation stemming from an over-reliance on organizations to self-regulate without external oversight. Their analysis also points to the ambiguity and inconsistent application of EU standards and criteria. 

In parallel, Laux et al. [23] explore the formation of public social trust in AI, concluding that it relies on the perception of the impartiality, neutrality, and autonomous governance of supervisory institutions rather than the direct assessment of risks. They argue that ensuring the perception of fairness and independence of the oversight entities is crucial for the AI Act to effectively build citizen trust in AISs.

### 3.2. Learned Intermediary Doctrine

In any case, within this regulatory landscape, medical device companies find a layer of protection from liability through the “**learned intermediary doctrine**”. This legal principle places a clear responsibility on physicians, emphasizing their duty to fully understand all potential risks associated with the use of medical devices because of their specialized knowledge, capability, and unique qualifications that can judiciously weigh the risks against the benefits of employing these devices for their patients. Therefore, failure to accurately assess and inform patients about the use of a medical device could serve as grounds for legal action by patients in instances of medical negligence [4]. 

The “*learned intermediary doctrine*” addresses how liability principles apply in the medical device context, focusing on patient interests. It designates **physicians as intermediaries** between the manufacturer and the end user, the patient, and prevents direct lawsuits against medical device manufacturers by patients because of the absence of any direct obligation from the manufacturers to them. In this framework, manufacturers fulfill their obligation to warn about product hazards by communicating these warnings to physicians. Subsequently, if the physician inadequately informs the patient about the inherent risks and advantages of the product, the responsibility for any ensuing liability rests with the physician [4]. 

According to strict product liability [24], producers are responsible for harm caused by defects in their products but tested and approved AI products that outperform their predecessors are unlikely to be labeled ‘defective’: strict liability could not usually be applied because a defect is something undetectable to a reasonable producer, as such a scenario would likely have prevented the product’s approval. 

Our AI-technology-based scenario is composed of a healthcare professional who has likely fulfilled his/her duty of care due to the technology’s high accuracy and reasonable reliance on its advice, and the producer of the technology does not bear any duty of care towards the patients. Consequently, even if the patient’s harm results from negligence by the technology’s producer, the producer is not liable for compensating the patient.

Furthermore, in the majority of instances, the prevailing legal doctrine is **negligence**, predicated upon fault, which stipulates accountability for damages resulting from a failure to exercise appropriate care. Liability can often be avoided by proving a lack of fault, especially in product liability, or by showing the defendant did not know about the risk. When machine learning technology indirectly aids human decision-making, rather than directly causing harm or damage, establishing negligence liability becomes increasingly challenging. 

### 3.3. System Transparency and “Black Box” Theory

In evaluating negligence liability for AI-driven decisions, it is essential to understand the decision-making process of AI. Enhancing AI **system transparency** could serve as an effective solution to address legal challenges and has already been recommended as a tool for regulation [24,25,26,27]. Ideally, this transparency would not only reveal how the AI operates but also explain the rationale behind its decisions, providing a comprehensive understanding of its reasoning process.

Technological systems that are difficult for humans to understand are often called ‘**black box**’ systems. In marketing, a “black box” represents the complex interaction between demand and supply. This concept also applies to artificial intelligence systems in healthcare, where the basic mathematical and logical operations are known, but how data are transformed through various steps is not as transparent. This “black box” issue subtends an eventual duty of medical professionals to explain the risks associated with a treatment strategy due to the fact that the diagnosis, prognosis, and treatment decisions are predicated on processes and determinations that are not transparent or understandable [3].

The black box features of deep learning and the lack of transparency regarding how results are obtained pose significant legal challenges. Sometimes, even the algorithms’ designers lack a complete understanding of how their AI processes data are produced, turning this lack of transparency essentially into a lack of knowledge [28].

It is important in the legal context to recognize that a system might be a ‘black box’ to one person but not to another. For example, the person who created a machine learning AI might explain how it makes decisions, but to a regular user, those processes might be completely opaque. This difference matters legally because the application of the law depends on what a person knew or should have known when a liability issue arose. Generally, the user only knows that they do not understand how the technology works, essentially viewing it as a ‘black box’. Their choice is limited to either trusting the technology’s decisions or not using them at all [24]. 

In medical AI systems, “opacity” can manifest in two main ways [29]. 

“Literal opacity” occurs when the AI’s decision-making process is completely hidden, even if algorithmic decision-making is clear, obscuring the connections between data and decisions. Meanwhile, “practical opacity” refers to a situation that is neither completely transparent nor entirely opaque, offering some insight into the AI’s operations but not full comprehension [30]. 

**Figure 4:**
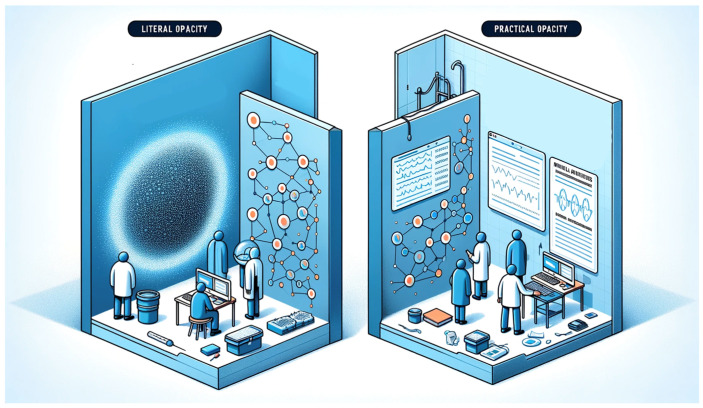
Literal opacity in AI obscures the entirety of its decision-making mechanisms, while practical opacity allows for partial visibility into the algorithmic processes. This image was generated with the assistance of DALL-E (by OpenAI).

These concepts, showed in Figure 4, are essential for recognizing the potential and limitations of medical AI in terms of accessibility and understandability.

Automation bias is a significant concern in the use of AI for diagnostic imaging. 

This bias involves prioritizing machine-generated diagnoses over those based on scientific knowledge and expertise, often disregarding human judgment or conflicting information, leading to *omission* or *commission* errors [31].

*Omission* errors happen when physicians, considering AI infallible, overlook or ignore mistakes generated by AI tools. High decision flow rates, where radiological examinations are reviewed quickly, increase the likelihood of this kind of error. These issues can be further exacerbated by AI making decisions based on features that are too nuanced for human detection. Conversely, *commission* errors occur when a machine’s decision is accepted or implemented despite contrasting evidence. The risks of automation bias are heightened in situations with a shortage of medical personnel, as there may be no radiologist available to verify the AI results [32]. 

This scenario is further complicated by the rapid development and premature application of AI techniques, causing user confusion to effectively incorporate this technology into their practice. Therefore, it is essential to promote updated regulatory policies and continuous education, as AI systems are becoming more widely available, complex, and powerful. Patients also need to be well-informed about the purposes, rights, and legal terms associated with AI in their health management [33]. The defining characteristics of actions undertaken by radiologists, in their professional capacity, involve autonomy in decision-making concerning both the choice of diagnostic services provided and the technical instruments employed, along with the personalized nature of the whole process. It is evident that these unique peculiarities necessitate harmonious integration with AI-driven automation [32,34].

The call for transparency gains relevance only when tailored to the needs of those who require an understanding of the AI’s decision-making processes. Hence, it is vital to define transparency in the context of the individuals seeking insight into how the AI operates: “transparency” means giving clear explanations in a way that makes sense to the target audience who need to understand.

In healthcare, AI algorithms need to be transparent and under strict oversight to clarify their decision logic and effects, ensuring explanations are patient-friendly [29]. 

This involves more than just making AI decisions understandable; it also requires clear communication about the algorithm’s limitations and its capability to protect patient identity and reduce harm [35,36,37]. 

In line with the previous discussion, the concept of Explainable Artificial Intelligence (XAI) is gaining significant attention in the scientific community [38,39,40]. XAI aims to ensure that algorithms and the decisions they produce are comprehensible to humans. It seeks to shift from the “black box” paradigm, where the internal workings of AI systems are obscure and opaque, to a “glass box” or “white box” approach, where transparency is the gold standard. Indeed, in a glass box model, full transparency represents the ideal scenario: every parameter is known, and the decision-making process is fully visible.

However, in many practical applications, particularly those involving complex models like deep learning, achieving complete transparency can be challenging. These models resemble a “translucent glass box”, with varying transparency levels, such as a window with different opacities, from clear to somewhat obscure. Lower opacity (or higher transparency) allows for a better understanding of the model, which can enhance trust in the AI system.

**Figure 5:**
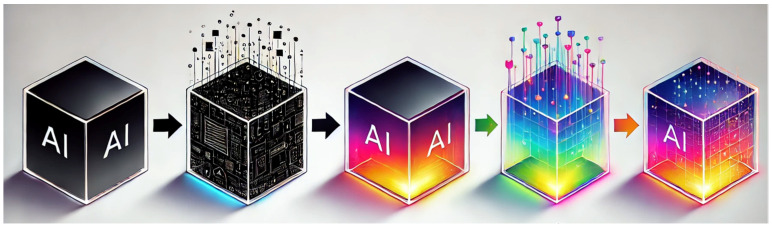
Here is an illustration depicting the evolution from the “black box” to “glass/crystal box” in AI, showcasing the concept of Explainable Artificial Intelligence (XAI) and enlightening the increasing transparency with a vibrant color transition. This image was generated with the assistance of DALL-E (by OpenAI).

In summary, as depicted in Figure 5, XAI seeks to transform AI systems into transparent, comprehensible tools, thereby encouraging greater trust and reliability. 

A highly accurate prediction model, like a “crystal ball”, may lose accuracy when simplified for transparency. Alternatively, if simpler steps are incorporated to enhance clarity, performance will be reduced. In healthcare, the priority often shifts towards accuracy, favoring the “crystal ball” approach. Consequently, in practical applications, full explainability is frequently compromised in favor of achieving higher accuracy and performance.

### 3.4. A New Healthcare Paradigm

A critical aspect of this process is determining how to effectively communicate AI-generated decisions to patients, aiming to integrate these insights into the collaborative decision-making framework, thereby directly influencing the doctor–patient relationship [41,42]. AI could reshape the traditional two-way doctor–patient relationship [29], introducing new players including AI systems, programmers, and product manufacturers. This shift might lead to a new form of decision-making process, defined as “*computernalism*”, where decisions may increasingly rely on computers and AI tools [43,44]. Actually, the evolution from the conventional bilateral dynamic to a trilateral “physician–computer–patient” relationship is already happening, marking a significant paradigm shift in healthcare [45]. 

Triberti et al. [46] suggested that this evolving relationship could lead to a “third wheel” effect, potentially compromising the efficacy of shared decision-making by positioning the physician as merely a mediator between the patient and the algorithm. This could lead to a shift in medical focus away from human skills [43], potentially heightening concerns over AI algorithms becoming so accurate that they replace critical medical judgment [47,48]. 

According to our opinion, the integration of AIS in daily practice could enhance the radiologist–patient relationship by allowing physicians to allocate more time for dialogue and interaction, thereby increasing patient trust.

Despite the remarkable capabilities of AI-based decision-making tools, it remains critical to keep the radiologist as the ultimate decision-maker, considering their comprehensive responsibility for clinical outcomes [49].

AI is evolving from an optional feature to a vital element of modern digital frameworks and avoiding AI use may be deemed unscientific and unethical. AI is set to enhance, merge with, or replace existing systems, initiating the “AI era in healthcare” [8,50]. In the event that AI-driven treatments surpass conventional approaches, they will not merely adjust but radically transform the benchmark for what constitutes ‘standard medical care’. This paradigm shift will necessitate that physicians acknowledge and fully utilize AI’s potential. Not embracing this new standard could lead to accusations of inadequate care, in light of AI’s proven efficacy. The debate on AI’s impact on the medical field focuses on how it will transform healthcare roles and tasks. This transition raises liability issues, especially when AI-assisted decisions cause harm. According to our research, medical practitioners are strictly liable for such outcomes, highlighting the need for clear regulations on the legal responsibilities of healthcare providers using AI. To minimize errors, AI decision-making processes and data should be regularly verified and updated. Additionally, patients should be informed about the use of AI in their care and its decision-making algorithms.

In the future, more than Artificial Intelligence, it would be more correct to think and talk about “Hybrid Intelligence” in the radiology field. Human intelligence would not be replaced but rather expanded by AI: this hybrid interaction could enhance both forms of intelligence by leveraging their complementary strengths.

## 4. Conclusions

The debate on how AI will reshape the medical field remains ongoing, with a significant focus on how it might transform the roles and tasks of healthcare professionals. This transition addresses the question of liability, particularly in scenarios where AI-assisted decisions lead to adverse outcomes. In such cases, the legal framework supports the fact that the overseeing medical practitioners are strictly liable, necessitating they compensate for any resultant harm or loss. This evolving scenario underscores the critical need for precise regulations on the legal obligations of healthcare providers who incorporate AI into their practice, ensuring that accountability is clearly established. Furthermore, to minimize the risk of errors, the decision-making processes and the underlying data used by AI systems should be subject to periodic verification and updates, ensuring their accuracy and relevance in a continuously evolving medical field. At the same time, patients must be informed when AISs are used in their care, along with an understanding of their decision-making algorithms.
